# Modifying the transition temperature, 120 K ≤ T_c_ ≤ 1150 K, of amorphous Fe_90−x_Co_x_Sc_10_ with simultaneous alteration of fluctuation of exchange integral up to zero

**DOI:** 10.1038/s41598-018-36891-2

**Published:** 2019-01-23

**Authors:** Y. N. Fang, H. Hahn, S. Kobe, R. Witte, S. P. Singh, T. Feng, M. Ghafari

**Affiliations:** 1 Herbert Gleiter Institute of Nanoscience, Nanjing University of Science and Technology, Nanjing, 210094 China; 2 Institute for Nanotechnology, Karlsruhe Institute of Technology (KIT), Hermann-von-Helmholtz- Platz 1, 76344 Eggenstein- Leopoldshafen, Germany; 3 Technische Universität Dresden, Institut für Theoretische Physik, D-01062 Dresden, Germany

## Abstract

Amorphous (a-) Fe_90−x_Co_x_Sc_10_ alloys have been produced by rapid quenching from the melt. The Curie temperature, T_C,_ was determined using both mean field theory and Landau’s theory of second-order phase transitions in zero and non-zero external fields. The dependence of T_C_ on the atomic spacing can be explained by the empirical Bethe-Slater curve. The value of T_C_ of a- Fe_5_Co_85_Sc_10_, determined by the above theoretical approaches is 1150 K, which is the highest T_C_ ever measured for amorphous alloys. The flattening of the measured normalized magnetization, M(T)/M(0), as a function of the reduced temperature, T/T_C_, is explained within the framework of the Handrich- Kobe model. According to this model the fluctuation of the exchange integral is the main reason for the flattening of M(T)/M(0). In the case of a-Fe_90_Sc_10_ without Co, however, the fluctuation of the exchange integral is dominant only at zero external field, B_ex_ = 0. At B_ex_ = 9 T, however, the fluctuation of the exchange integral has no conspicuous effect on the reduction of the magnetization. It is shown that at B_ex_ = 9 T the frozen magnetic clusters control the behaviour of the reduced magnetization as function of T/T_C_. In contrast to other ferromagnetic alloys, where the flattening of M(T)/M(0) is characteristic for an amorphous structure, the a- Fe_5_Co_85_Sc_10_ does not exhibit any trace of the fluctuation of the exchange integral.

## Introduction

Efforts have been made to produce soft magnetic materials with high saturation magnetization, high Curie temperature, low magnetic coercivity, high permeability and low magnetostriction. These aims are mostly achieved by Co-based amorphous alloys. These physical properties combined with outstanding soft magnetic properties and high crystallization temperature is of central importance for the application in many technical devices^1^. Currently, it has been reported that a-Co_90_Sc_10_ has a high Curie temperature (Tc > 860 K) and good soft magnetic properties^2^.

This investigation is based on objectives aimed at producing amorphous alloys with high transition temperature and simultaneous reduction of variations of magnetization curve as a function of temperature to approximately zero in the range T = 300 ± 100 K. Additional major goal of this investigation is to study under which conditions the fluctuation of exchange integral becomes extinct. The amorphous transition metal-rich Fe_90−x_Co_x_Sc_10_ system is one of the means to achieve the above mentioned objective. In addition, the amorphous transition metal-rich Fe_90−x_Co_x_Sc_10_ system, which exhibits high values of the Curie temperature and the magnetization is of interest in order to study the fundamentals of the dependence of the magnetization on the Co concentration.

**Figure 1:**
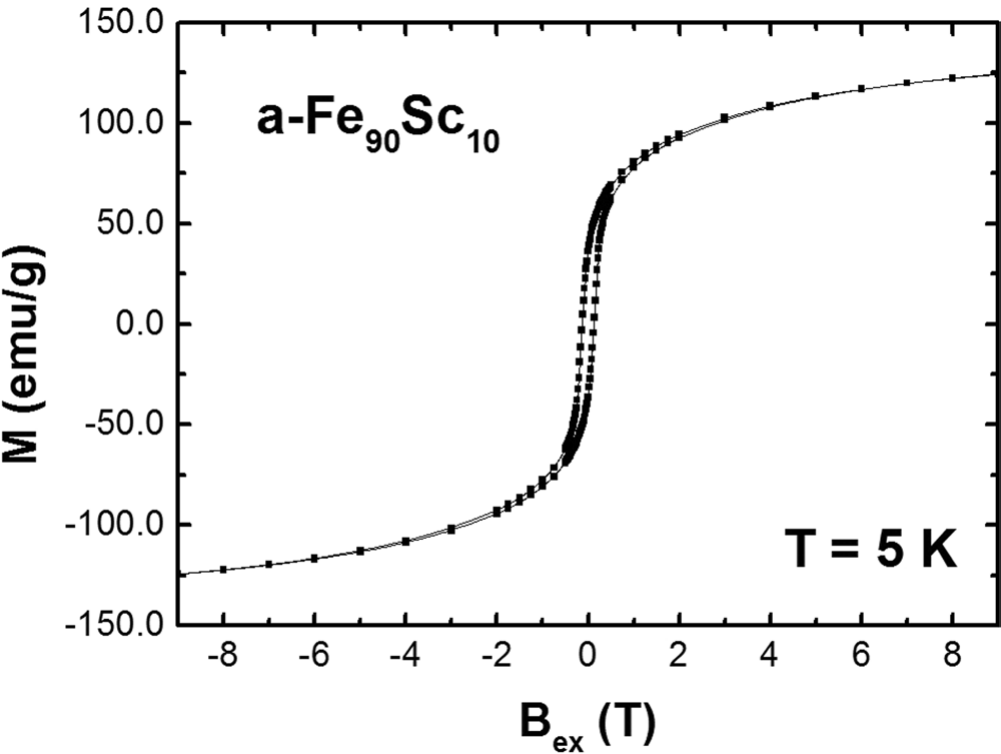
Magnetization of a- Fe_90−_Sc_10_ as a function of B_ex_ at T = 4.2 K. The a- Fe_90−_Sc_10_ is magnetically disordered. The saturation magnetization cannot be achieved even at B_ex_ = 9 T (see text). The magnetic transition is T = 120 K.

In contrast to a-Co_90_Sc_10_ alloys^2^, a-Fe_90_Sc_10_ alloys are magnetically harder with a coercivity of about H_c_ ≈ 0.18 T, Fig. 1. The magnetization of a-Fe_90_Sc_10_ at a temperature of 5 K cannot be saturated even at applied external fields B_ex_ ≥ 8 T, Fig. 1. On the other hand, the magnetic transition temperature (T_C_ = 120 K) is extremely reduced in comparison to the amorphous Co based alloys. The physical reason for this abnormal magnetic behaviour of based on frustration of exchange coupling and has been reported in ref.^3^.

Combining the different magnetic behaviour of the two amorphous systems CoSc and FeSc was the initial idea behind synthesizing amorphous ternary alloys in the CoFeSc system.

## Experimental

**Figure 2:**
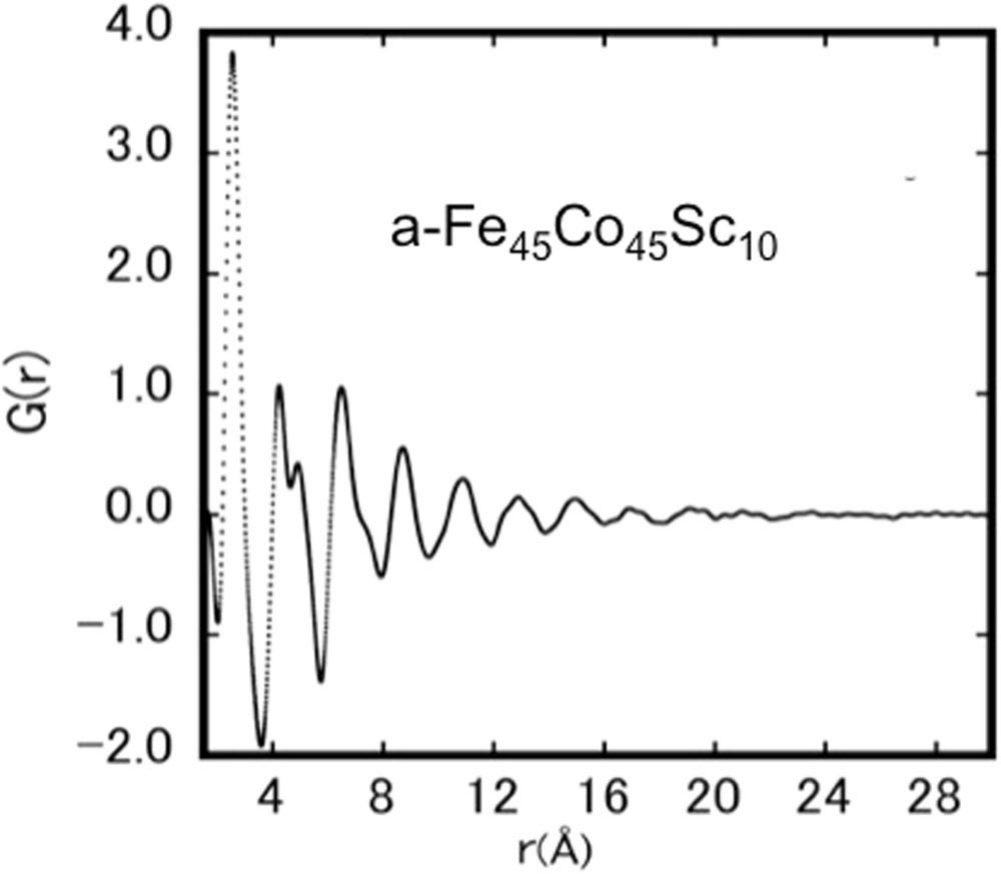
A representative Pair Distribution Functions, G(r), of a-Fe_90−x_Co_x_Sc_10_ at room temperature.

Master alloys with the nominal composition of Fe_90−x_Co_x_Sc_10_ (0 ≤ < x < ≤ 90) were synthesized by arc melting in an argon atmosphere. Amorphous Fe_90−x_Co_x_Sc_10_ ribbons were prepared using melt spinning with a wheel speed of 45 m/s in an argon atmosphere. The amorphous ribbons had a thickness of approx. 30 μm and a width of approx. 2 mm. Energy Dispersive X-ray Spectroscopy EDX (Oxford Instruments) was used to analyze the composition of the ribbons in comparison to the starting material. X-ray diffraction measurements of a-Fe_90−x_Co_x_Sc_10_ allow to determine the amorphous structure and under certain conditions to determine the atomic structure and the inter-atomic distances, leading to radial distribution functions. A high-flux rotating anode X-ray diffractometer with high-resolution parallel beam optics was employed to confirm the amorphous structure of the ribbon samples. The selected photon wavelength of Mo-radiation was λ_Mo-Kα1_ = 0.7107 Å. The conversion of the X-ray diffraction data of a- Fe_90−x_Co_x_Sc_10_ to atomic Pair Distribution Function, PDF, was made using the PDFgetX3 software^4^. To proof the reliability of this method, the PDF of two representative samples (a-Fe_90_Sc_10_ and a-Co_90_Sc_10_) measured and evaluated^2,5^ at a synchrotron source (Spring-8) with a substantially smaller photon wavelength of 0.2 Å was compared with those obtained by the method described above. It could be confirmed that the data measured using the laboratory instrument coincide well with those measured at the synchrotron, enabling the use of Mo-source instrument with better accessibility. The PDF of a representative sample, a- Fe_45_Co_45_Sc_10_, is shown in Fig. 2. It is evident that the atomic structure of the ternary amorphous alloy is similar to that of a-Fe_90_Sc_10_ or a-Co_90_Sc_10_ both reported earlier to exhibit a distorted bcc structure^6^. It was shown that the distorted bcc structure describes also the atomic structure of other compositions x over the entire compositional range^6^. In contrast to the crystalline Fe_100−x_Co_X_ alloys^7^, a-Fe_90−x_Co_x_Sc_10_ alloys show no structural changes as a function of composition of Co. It is important to note that the partial distribution functions allow to access information such as the distribution and positions of nearest neighbours and thus provide a base for the discussion of magnetic behaviour of a-Fe_90−x_Co_x_Sc_10_ alloys.

The magnetic studies were performed using SQUID- Quantum Design as well as a Physical Property measurement System (PPMS®)- Quantum Design in temperature range 4.2 K ≤ T ≤ 1000 K with external magnetic fields 0 < B_ex_ ≤ 9 T.

For discussion regarding the magnetic behaviour as a function of temperature, it is important that magnetic saturation is achieved. In the case of a-Fe_90_Sc_10,_ it is, however, difficult to align the spin in external fields directions. The detail information for this behaviour is given in ref.^3^. The internal magnetic hyperfine field splitting, B_hf_, is in the most transition metal based alloys proportional to the magnetic moment of Fe^8^. Fortunately, the B_hf_ is unrestricted to the orientation of spins and it is assessable by nuclear methods such as Mössbauer spectroscopy. As with most transition metals^8^, assuming that the following equation, M(T)/M(0) = B_hf_(T)/B_hf_(0), is authentic between the magnetization and average magnetic hyperfine field, the magnetic behaviour as a function of temperature of a- Fe_90_Sc_10_ will be discussed in this report. The details of the evaluation of B_hf_(T) have been discussed in ref.^9^.

For x > 0, the Curie temperatures of a- Fe_90−x_Co_x_Sc_10_ were verified through two different methods: (1) for x ≤ 35, T_C_ was deduced by mean of Landau theory^10^. (2) For x > 35, the mean field theory were applied to calculated the T_C_. The reason for the choice of two different methods is the crystallization of samples. At x > 35, the T_C_ is higher than the crystallization temperature and the Landau theory cannot be applied^10^.

In general, T_C_ is a second order phase transition from magnetically random disordered state to a magnetically well-ordered state. According to Landau^10^ the fourth-order expansion of free minimum energy in an external field or in zero external field at T_C_ delivers a point of inflection for the slope of M(T)/M(0) at t = (T − T_C_)/T_C_ = 0.

**Figure 3:**
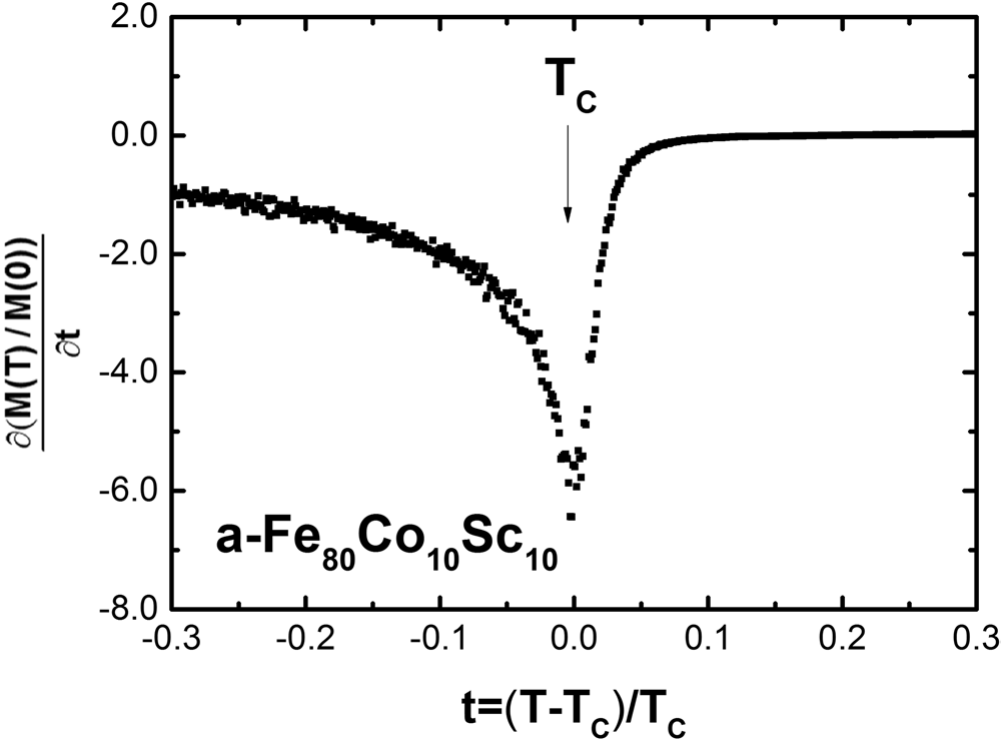
A representative plot of the slope of M(T)/M(0) as a function of normalized temperature t = (T − T_C_)/T_C_ is presented. According to Landau theory is the Curie temperature at the point of inflection, t = 0 (See text).

A representative figure of the slope, ∂M(T)/∂M(0), as a function of t = (T − T_C_)/T_C_ is presented in Fig. 3.

At x > 35, T_C_ is above crystallization temperature T_X_. For determination of T_C,_ the measured magnetization in the range, 90 > x > 35, and at T < T_c_ were satisfactorily matched to the mean field theory equation:

1 $$\frac{{\rm{M}}({\rm{T}})}{{\rm{M}}(0)}=\,\tanh [\frac{{\rm{M}}({\rm{T}})/{\rm{M}}({\rm{0}})}{{\rm{T}}/{{\rm{T}}}_{{\rm{C}}}}]$$

The results will be discussed in the next section.

**Figure 4:**
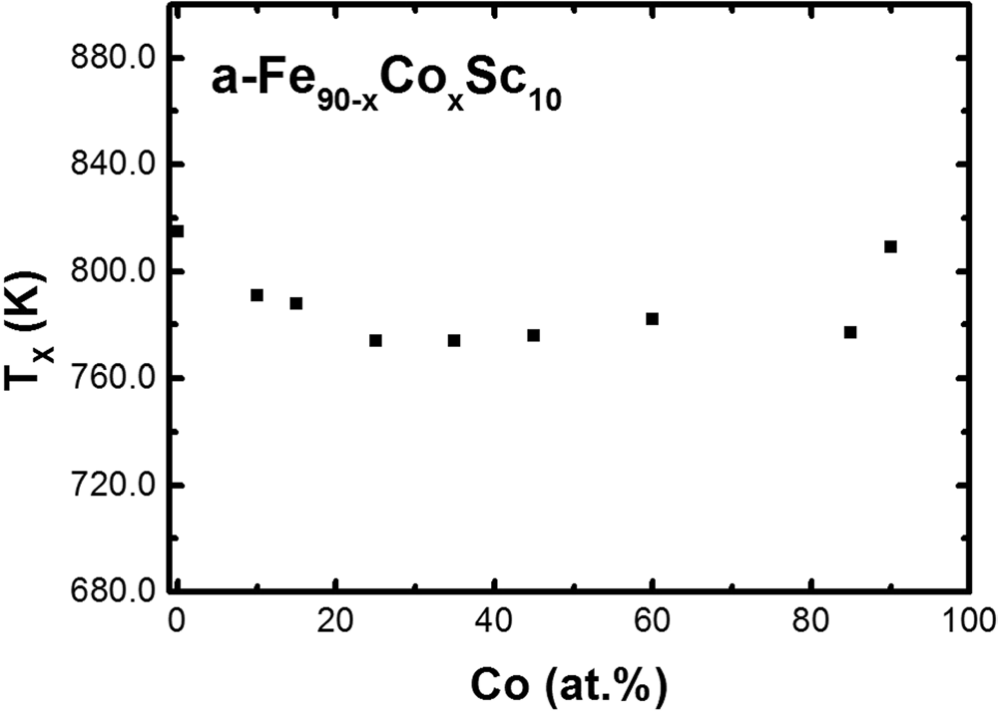
Crystallization temperature as a function of Co.

The determination of the crystallization temperature, T_X_ of a- Fe_90−x_Co_x_Sc_10_ were performed with a Differential Scanning Calorimetry, NETZSCH STA 449F3 at a heating rate of 20 K/min, Fig. 4.

## Results and Discussions

### Curie temperature

FeCo-based amorphous alloys have among all transition metals based ferromagnetic amorphous alloys the highest Curie temperature^2^.

**Figure 5:**
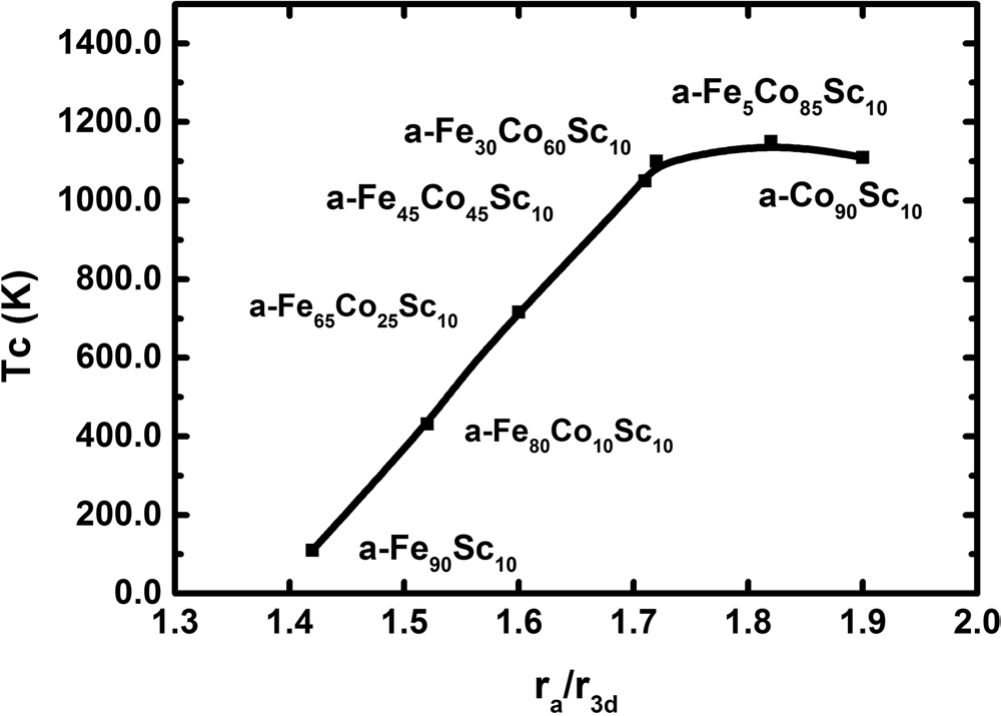
Curie temperature as a function of the ratio of r_a_/r_3d_. r_a_ and r_3d_ are denoted as the first maximum at PDF-curve and 3d radii respectively.

The highest Curie temperature is, however, low in comparison to the pure crystalline transition metals. The reasons for this behaviour are: (a) Different arrangements of short-range order in comparison to the crystalline counter part. (2) Alloying effect (3) Distribution of the interatomic distances (4) Different electron structure^11^. Recently, it has been reported that a- Co_90_Sc_10_^2^ has the highest T_C_ ≈ 1000 K among the amorphous transition metals. One reason for this high T_C_ is the structure of this alloy^6^. Amorphous Transition-metal rich Sc alloys have a distorted bcc structure^6^. According to the experimental Slater- Pauling curve^12,13^, the ratio of r_a_/r_3d_ determine the exchange integral and with that the T_C_. r_a_ and r_3d_ are denoted as atom diameters and 3d shell radii, respectively. Hence, it should be possible to increase the T_c_ toward higher values by altering the ratio r_a_/r_3d_. For this purpose, Fe replaced the Co atoms in the amorphous Co_90_Sc_10_ alloy. The resulting increase of T_c_ is shown in Fig. 5. The atomic diameter, r_a_, is selected from the first maximum of PDF curve. The resulting curve follows the experimental Slater-Pauling curve. Contrary to the crystalline Fe_100−x_Co_x_, there are no structural transitions in a- Fe_90−x_Co_x_Sc_10_. The shape of the resulting PDF is similar for all a- Fe_90−x_Co_x_Sc_10_. The main variation is the atomic distances. Using the experimental idea of Slater-Pauling^12,13^, it was possible to reach a T_C_ at 1150 K. This finding is of special interest for the use of soft magnetic properties.

### Magnetization

**Figure 6:**
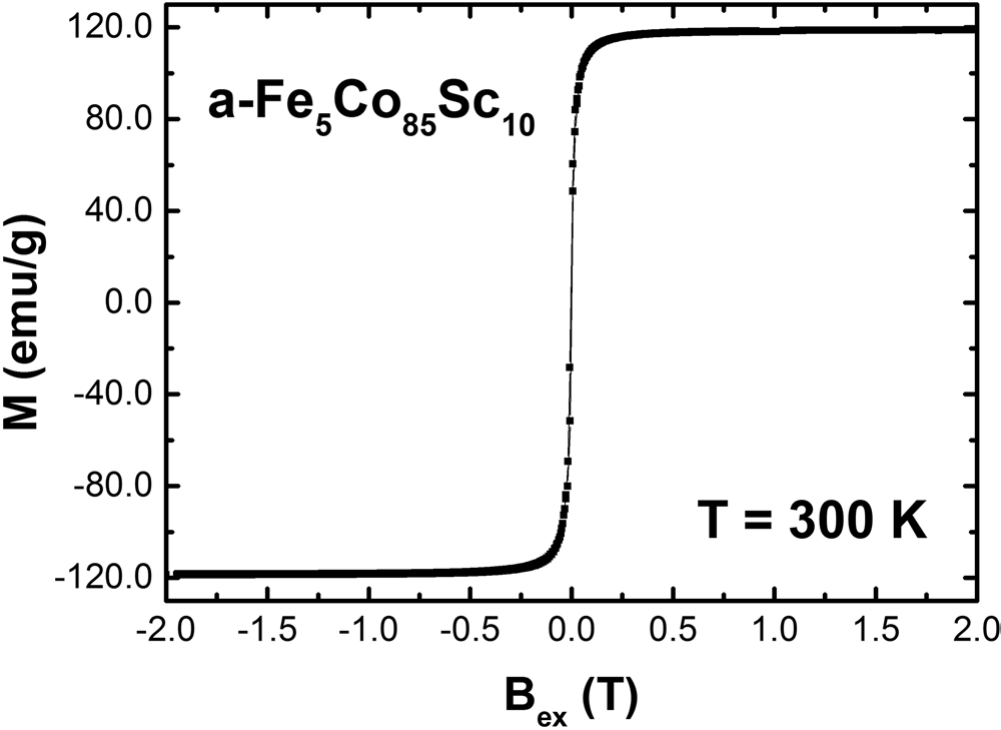
Magnetization as a function of external fields of a- Fe_85_Co_5_Sc_10_ at T = 300 K.

Amorphous samples, a-Fe_5_Co_85_Sc_10_, are magnetically soft with a corresponding magnetic moment of the transition metals (Fe and Co) of about 1.4 μ_B_ at a temperature of 300 K, as seen in Fig. 6. Replacing just 5% of the Co-atoms by Fe results in an increase of the Curie temperature to 1150 K for a- Fe_5_Co_85_Sc_10_, which is, to the best of our knowledge, the highest T_C_ ever obtained for any amorphous alloys. Furthermore, the magnetic moment of a- Fe_5_Co_85_Sc_10_ is higher than a-Co_90_Sc_10_.

### Magnetization curve

**Figure 7:**
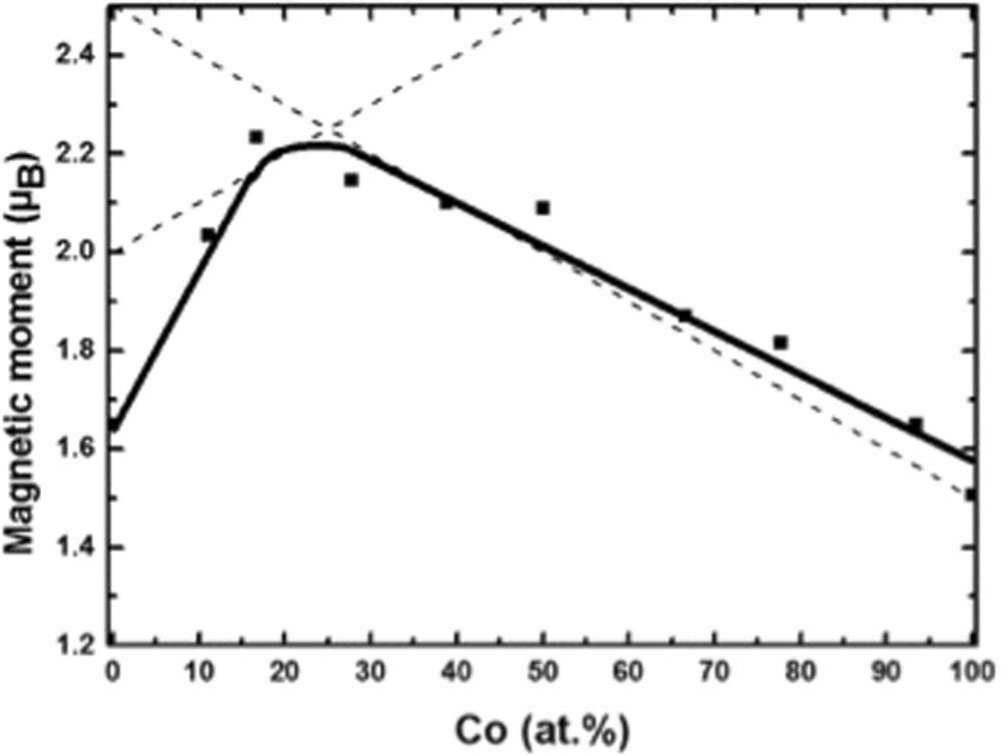
Magnetic moment as a function of Cobalt measured at 4.2 K. The straight dashed lines present calculated values (See text).

As mentioned, a- Fe_90−x_Co_x_Sc_10_ has a distorted bcc structure. The experimental observed magnetic moment as a function of the Co content at 4.2 K is presented in Fig. 7. A maximum of magnetic moment is indicated at about x = 25. The behaviour of M(T) as a function of Co contents is similar to one reported for the crystalline bcc Fe-Co alloys. Using self- consistent local spin-density functional calculation, Schwarz *et al*.^14^ have shown that in the case of bcc FeCo alloys are the itinerant 3d-electrons band with 3d spin up as well as 3d spin down intensely concentrated close to Co and Fe atoms. There is, however, a minor negative contribution of about −0.02 µ_B_ between atoms. This contribution is due to the p-electrons. The resulting of Magnetic Compton X-ray Scattering^2,15^ shows, however, a negative value of about −0.23 µ_B_ and −0.49 µ_B_ for a-Co_90_Sc_10_ and bcc-Fe, respectively. Similar to crystalline Fe_1−x_Co_X_ alloys, the shape of magnetization curve of a- Fe_90−x_Co_x_Sc_10_ consists of two physical different appearances^14^: (1) At 100 < x < 25, the spin up electrons are filled. An increase of Fe causes a decrease of spin down and with that an increase of the magnetic moment as appeared in Fig. 5^14,16^. Using the equation suggested by Schwarz *et al*.^11,16,17^: M = 2 N − Z with N = 5.22 was an agreement reached. The values, M, N and Z are defined as magnetic moment, average number of spins and number of valence electrons per atom, respectively. The value of N is slightly smaller than that chosen for bcc Fe^14^, Fig. 7. For x < 25, an agreement cannot be achieved satisfactorily between theory and experiment, because the Fe atoms are in a-Fe_90_Sc_10_ magnetically disordered. They experience positive, ferromagnetic, as well, negative antiferromagnetic interactions^3^.

(2) At x > 25, the spin downs are pinned^14^ and a decrease of Fe leads to an increase of magnetic moment according to the equation: M = Z-2N. Choosing a value: N = 3.125 lead to good agreement between experimental data and the suggested equation as presented in Fig. 7. The selected N is in comparison to bcc-Fe higher. This could be originated in additional filling of spin down band structure by Sc atoms.

### Reduced Magnetization: M(T)/M(0)

**Figure 8:**
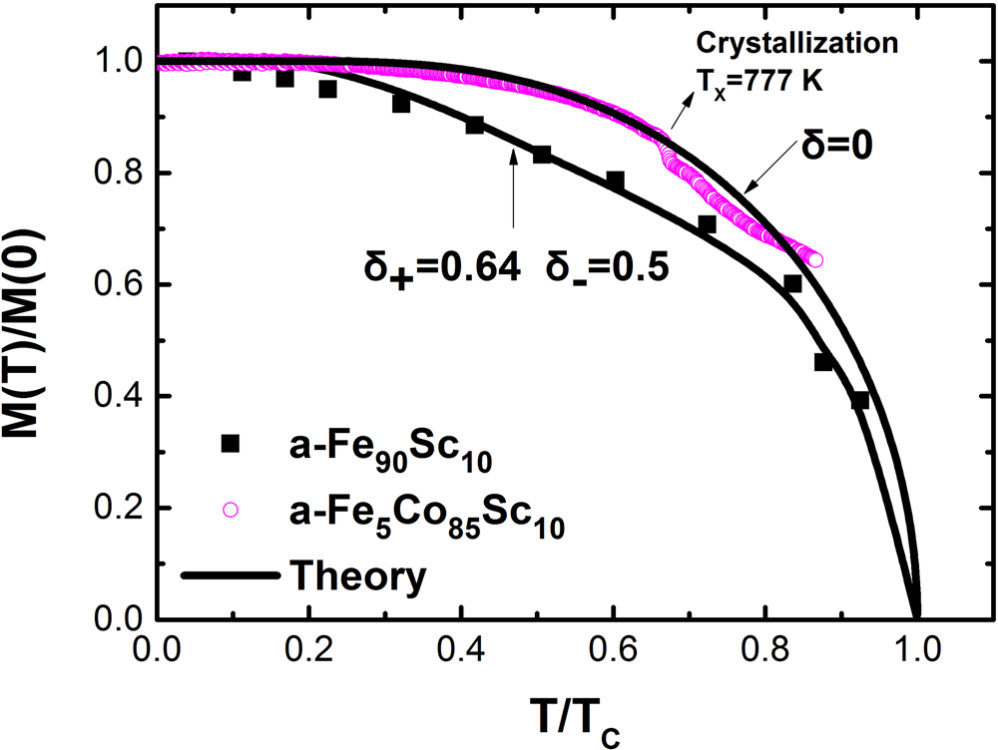
Reduced magnetization M(T)/M(0) as a function of reduced temperature T/T_C_ for two representative samples. The curves are fitted with asymmetric exchange integral fluctuation according to ref.^22^.

The reduced saturation magnetization, M(T)/M(0), of a-Co_85_Fe_5_Sc_10_ as a function of reduced temperature, T/Tc, measured from 4.2 K up to the crystallization temperature, T_X_ = 777 K, agrees with the prediction of the mean field theory, Fig. 8. This behavior is comparable to crystalline materials. In contrast to other distorted systems such as amorphous alloys no modification of the Brillouin function was necessary to calculate the experimental data. The best agreement of the theoretical description with the experimental data is achieved by using an extrapolated Curie temperature, T_C_ = 1150 K. As crystallization of the amorphous structure sets in prior to reaching the Curie temperature, the transition temperature cannot be determined experimentally. The same calculation of the Curie temperature has been employed to determine T_C_ for crystalline Fe, Co and Ni^18^.

According to Handrich and Kobe, the mean field theory predicts successfully the temperature dependence of the reduced saturation magnetization, M(T)/M(0), if the Brillouin function is modified^19–21^. The modified Brillouin function takes into account the effect of the distorted structure and the variation of interatomic spacing between nearest neighbors on exchange integral. In Handrich and Kobe theory^19–21^, the reduced saturation magnetization is given by:

2 $$\frac{{\rm{M}}({\rm{T}})}{{\rm{M}}(0)}=\frac{{\rm{1}}}{{\rm{2}}}\{{{\rm{B}}}_{{\rm{s}}}[(1+{\rm{\delta }}){\rm{x}}]+{{\rm{B}}}_{{\rm{s}}}[({\rm{1}}-{\rm{\delta }}){\rm{x}}]\}$$

x and δ are defined as:

$${\rm{x}}=\frac{3{\rm{S}}}{{\rm{S}}+1}\,\frac{{\rm{M}}({\rm{T}})}{{\rm{M}}(0)}\,\frac{{{\rm{T}}}_{{\rm{C}}}}{{\rm{T}}},\,{\rm{\delta }}=\sqrt{\langle {{\rm{\Delta }}J}^{2}\rangle /{\langle {\rm{J}}\rangle }^{2}}$$

The parameters B_s,_ J and ΔJ are defined as the Brillouin function, exchange integral and exchange fluctuation, respectively.

According to Gallagher *et al*.^22^, the Handrich- Kobe model would be improved, if the asymmetric exchange fluctuations were included. The asymmetrical distribution of the exchange integral is the result of the form of the empirical Bethe-Slater curve, which permits the replacement of δ parameters in equation (2) by two different parameters, δ_+_ and δ_−_. The alternative equation is expressed as:

3 $$\frac{{\rm{M}}({\rm{T}})}{{\rm{M}}(0)}=\frac{1}{2}\{{{\rm{B}}}_{{\rm{s}}}[({1+{\rm{\delta }}}_{+}){\rm{x}}]+{{\rm{B}}}_{{\rm{s}}}[({\rm{1}}-{{\rm{\delta }}}_{-}){\rm{x}}]\}$$

Using the method of Gallagher *et al*.^22^ a zero exchange fluctuation parameter, δ = √(ΔJ2)/(J)^2^ = 0, was deduced for a-Fe_5_Co_85_Sc_10_. The best agreement between the theory, equation 3, and measurements of a-Fe_90_Sc_10_ was reached by an asymmetric exchange fluctuation parameters, δ+ = 0.64 and δ- = 0.5, Fig. 8. In the case of a-Fe_90_Sc_10_ the saturation magnetization cannot be reached in external fields. Therefore for the determination of M(T)/M(0), we must move away from the standard ways such as SQUID- or PPMS measurements. Mössbauer effect is an alternative method for the determination of M(T)/M(0). As discussed in section 2, the measured average magnetic hyperfine field, B_hf_(T), at zero external magnetic field is in transition metals proportional to the M(T). Therefore for the calculation of M(T)/M(0), the equation: B_hf_(T)/B_hf_(0) = M(T)/M(0) has been used. It is worth remembering that the a-Fe_90_Sc_10_ consists of magnetic clusters with an average sizes of about 100 atoms. The measured B_hf_(T)/B_hf_(0) = M(T)/M(0) is within clusters.

In the next section, it will be shown that for a-Fe_90_Sc_10_, the exchange fluctuation parameter in an applied magnetic field of 9 T can be depressed to zero. In this case the magnetic movement of whole single cluster and their interaction with other clusters are decisive for the behavior of M(T). The individual spins in the cluster do not play an essential role. This uncommon effect is of special interest and will be discussed  in details.

The M(T)/M(0) curve of ferromagnetic amorphous alloys as a function of T/T_C_ are in contrast to the comparable crystalline alloys flatter. The mean field theory seems unable to explain the behaviour of magnetization as a function of temperature. However, by considering the distorted short-range order of atoms, Handrich and Kobe^19,20^ were able to explain the magnetization behaviour as function of temperature in the context of a modified mean field theory. A meaningful improvement on the theory of Handrich and Kobe were reached by considering the asymmetric exchange fluctuation of exchange integral according to Bethe-Slater curves. Using equation 4 in ref.^22^, the measured M(T)/M(0),or average magnetic hyperfine field B_hf_(T)/B_hf_(0), were matched to the theory. The results for two representative samples are presented in Fig. 8.

**Figure 9:**
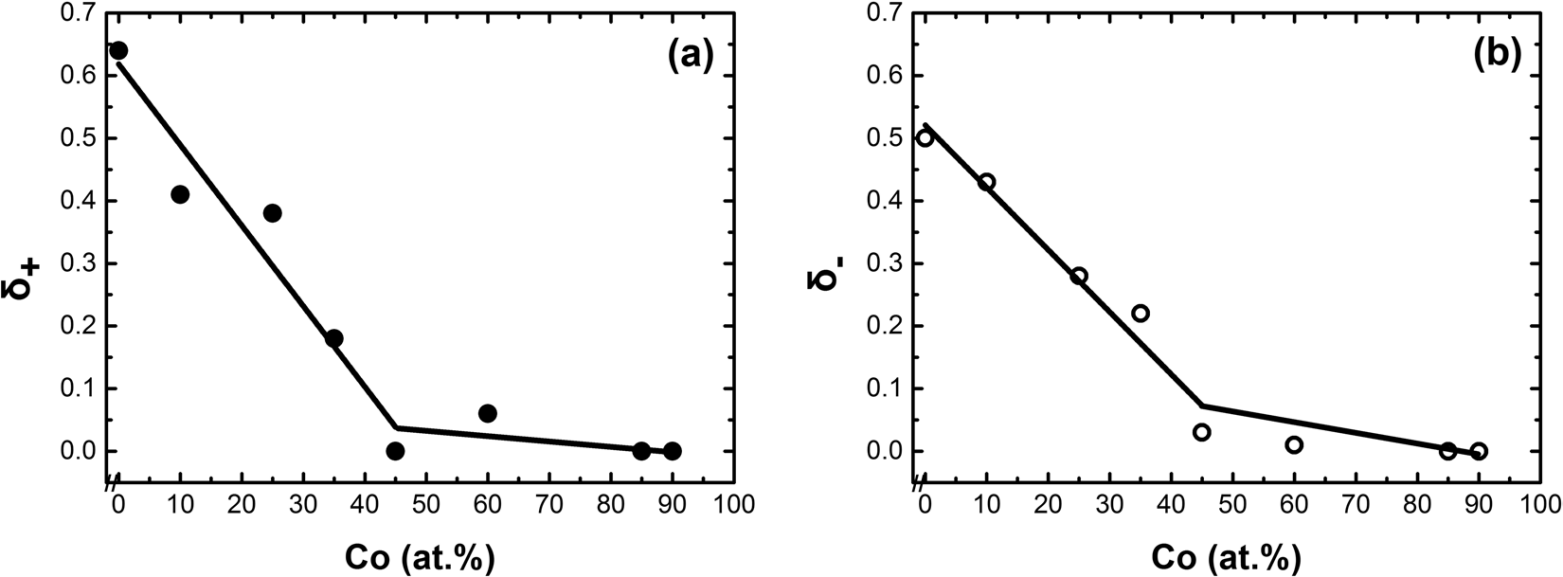
(**a**,**b**) Asymmetric exchange integral fluctuations, δ_+_ and δ_−_, as a function of Cobalt. δ_+_ and δ_−_ were determined from the best agreement between theory and experiment (see text).

The two asymmetric parameters, .δ_+_.δ_−_ of a- Fe_90−x_Co_x_Sc_10_ are presented in Fig. 9. The two asymmetric parameters are in a comparative agreement to the Bethe-Slater curve. Above x > 25, a variation of exchange integral is rather small; therefore the δ_+_ and δ tend to be low as presented in Fig. 9.

### M(T)/M(0) of a-Fe_90_Sc_10_

a-Fe_90_Sc_10_ is an exception: This amorphous alloy consists of ferro- and antiferromagnetic couplings. In addition, this alloy is made up of separated magnetic clusters with an average size of about 10 to 12 Å^3^ with a transition temperature at 120 K in zero field^3^. At T < 120 K, the spins have inside the clusters a mixed coupling (ferro- and antiferromagnetic). The resulting reduced magnetization M(T)/M(0) as a function of T/T_C_ inside the clusters can be described with the modified Brillouin function according to Handrich and Kobe theory. The outcome of the calculation and the measurements inside the clusters are presented in Fig. 8.

A totally different outcome was registered in the temperature dependence of M(T)/M(0) at external magnetic field, B_ex_ = 9 T.

**Figure 10:**
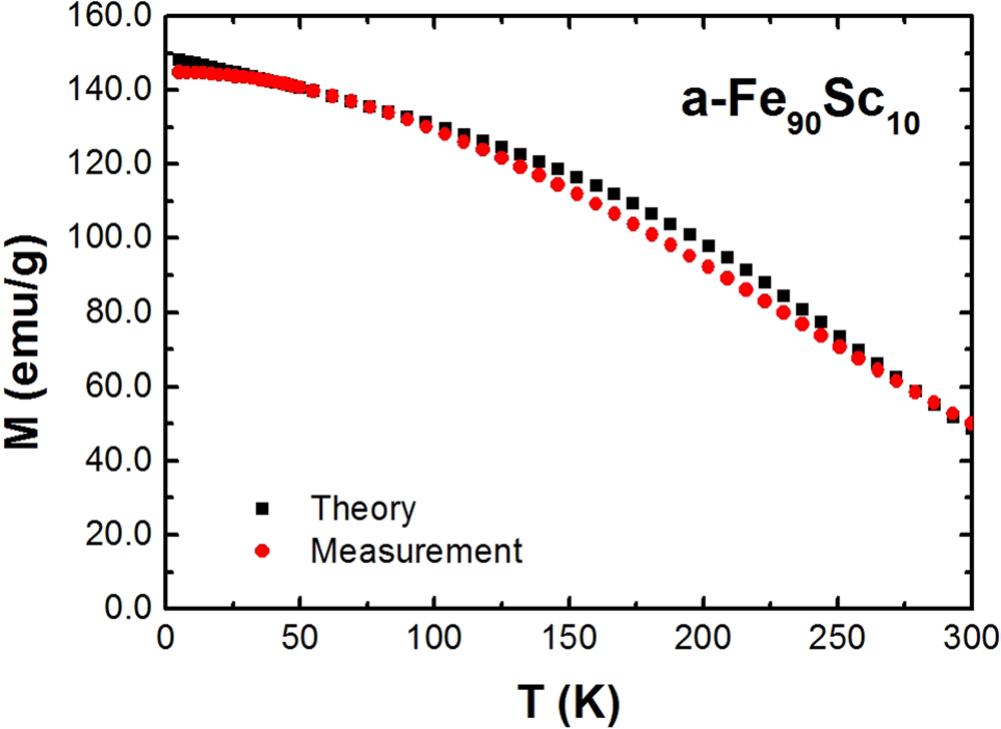
Measured and calculated magnetization as a function temperature of a-Fe_90_Sc_10_ measured at B_ex_ = 9 T (see text).

In a-Fe_90_Sc_10_, the magnetic clusters are relative to each other randomly distributed and there is no magnetic interaction between them. In other words, the clusters behaviour to each other likes individual atoms in paramagnetic state. Using an average magnetic moment of 100 µ_B_ for every cluster, a good agreement between experiment and mean field theory of paramagnet can be achieved. In this case the exchange fluctuations have no influence on M(T)/M(0) of clusters. The results are presented in Fig. 10.

## Conclusions

Amorphous Fe_90−x_Co_x_Sc_10_ alloys have been prepared by rapidly quenching the melt. The short range order, the magnetic moment, the Curie temperature and the temperature dependence of magnetization have been investigated in detail. The results obtained for amorphous transition rich-Sc alloys agree with the prediction of modified mean field theory. The amorphous Fe_5_Co_85_Sc_10_ alloys follow up to crystallization theory the exact mean field theory without any modification. Above x > 35 K, the magnetic moment can be explained satisfactorily with the theoretical band model of bcc structure. The calculated T_C_ of 1150 K of a- Fe_5_Co_85_Sc_10_ is the highest T_C_ reported so far for amorphous alloys. The exceptional magnetic character of an amorphous Fe_90_Sc_10_ alloy is discussed in the framework of magnetic clusters with distorted bcc structure.

Hence we are led to conclude that the high Curie temperature is related to the bcc ordering in these alloys.
